# Supplementary material for: Homeostatic regulation through strengthening of neuronal network-correlated synaptic inputs
Source: eLife. 2022 Dec 14;11:e81958. doi: 10.7554/eLife.81958 (PMC9803349; doi:10.7554/eLife.81958)
Supplement: Figure 1—source data 1. [file elife-81958-fig1-data1.docx]

| **Statistical Comparisons**  **for Figure 1** | | | **Comparison** | **Result** | |
| --- | --- | --- | --- | --- | --- |
| **Panel** | **Description** | **Test** |  | **p value** | **n value** |
| **1D** | % of inactive spines  Control vs Deprived | *Chi-square*  *test* | +12 hrs: Con (0.01 %) vs Dep (19.7 %) | p < 0.001 | Deprived = 434 spines  Control = 648 spines  Deprived + TNF-α inhibitor = 272 spines |
|  |  |  | +24 hrs: Con (3.8 %) vs Dep (12.4 %) | p < 0.001 |  |
|  |  |  | +48 hrs: Con (2.3 %) vs Dep (10.1 %) | p < 0.001 |  |
| **1F** | Normalized change in amplitude of spine events for all spines  Deprived  vs  Control  vs  Deprived + TNF-α inhibitor | *Two-Way ANOVA with post-hoc test* | Control vs Deprived | p < 0.001 |  |
|  |  |  | -24 hrs: Con vs Dep | p = 0.917 |  |
|  |  |  | -1 hrs: Con vs Dep | p = 0.917 |  |
|  |  |  | +12 hrs: Con vs Dep | p = 0.571 |  |
|  |  |  | +24 hrs: Con vs Dep | p < 0.001 |  |
|  |  |  | +48 hrs: Con vs Dep | p < 0.001 |  |
|  |  |  | Deprived+TNF-α inhibitor vs Deprived | p < 0.001 |  |
|  |  |  | -24 hrs: Dep+TNF-α inhibitor vs Dep | p = 0.936 |  |
|  |  |  | -1 hrs: Dep+TNF-α inhibitor vs Dep | p = 0.936 |  |
|  |  |  | +12 hrs: Dep+TNF-α inhibitor vs Dep | p = 0.005 |  |
|  |  |  | +24 hrs: Dep+TNF-α inhibitor vs Dep | p = 0.018 |  |
|  |  |  | +48 hrs: Dep+TNF-α inhibitor vs Dep | p < 0.001 |  |
|  |  |  | Control vs Deprived+TNF-α inhibitor | p = 0.661 |  |
|  |  |  | -24 hrs: Con vs Dep+TNF-α inhibitor | p = 0.959 |  |
|  |  |  | -1 hrs: Con vs Dep+TNF-α inhibitor | p = 0.959 |  |
|  |  |  | +12 hrs: Con vs Dep+TNF-α inhibitor | p = 0.005 |  |
|  |  |  | +24 hrs: Con vs Dep+TNF-α inhibitor | p = 0.019 |  |
|  |  |  | +48 hrs: Con vs Dep+TNF-α inhibitor | p = 0.415 |  |

**Figure 1-source data 1.** Statistical comparisons for Figure 1.
