# Supplementary material for: Homeostatic regulation through strengthening of neuronal network-correlated synaptic inputs
Source: eLife. 2022 Dec 14;11:e81958. doi: 10.7554/eLife.81958 (PMC9803349; doi:10.7554/eLife.81958)
Supplement: Figure 1—figure supplement 1—source data 1. [file elife-81958-fig1-figsupp1-data1.docx]

| **Statistical Comparisons**  **for Figure 1 - figure supplement 1** | | | **Comparison** | **Result** | |
| --- | --- | --- | --- | --- | --- |
| **Panel** | **Description** | **Test** |  | **p value** | **n value** |
| **1-1E** | Normalized change in frequency of spine events for  all spines  Control vs Deprived | *Two-Way ANOVA with post-hoc test* | Frequency: Control vs Deprived | p < 0.001 | Deprived = 434 spines  Control = 648 spines |
|  |  |  | -24 hrs: Con vs Dep | p < 0.001 |  |
|  |  |  | -1 hrs: Con vs Dep | p < 0.001 |  |
|  |  |  | +12 hrs: Con vs Dep | p < 0.001 |  |
|  |  |  | +24 hrs: Con vs Dep | p < 0.001 |  |
|  |  |  | +48 hrs: Con vs Dep | p < 0.001 |  |
|  |  | *One-Way*  *Repeated measures ANOVA* | Frequency: Dep -1 hrs vs Dep 12,24,48 hrs | p = 0.229 |  |
|  |  |  | Frequency: Con -1 hrs vs Con 12,24,48 hrs | p = 0.009 |  |
|  |  |  | Con -1 hrs vs +12 hrs | p < 0.001 |  |
|  |  |  | Con -1 hrs vs +24 hrs | p = 0.268 |  |
|  |  |  | Con -1 hrs vs +48 hrs | p = 0.002 |  |
| **1-1F** | Correlation normalized frequency and amplitude | *Pearson correlation* | All spines (r = -0.09) | p = 0.813 | Deprived = 434 spines |

**Figure 1-source data 2.** Statistical comparisons for Figure 1 - figure supplement 1.
