# Supplementary material for: Homeostatic regulation through strengthening of neuronal network-correlated synaptic inputs
Source: eLife. 2022 Dec 14;11:e81958. doi: 10.7554/eLife.81958 (PMC9803349; doi:10.7554/eLife.81958)
Supplement: Figure 2—source data 1. [file elife-81958-fig2-data1.docx]

| **Statistical Comparisons**  **for Figure 2** | | | **Comparison** | **Result** | |
| --- | --- | --- | --- | --- | --- |
| **Panel** | **Description** | **Test** |  | **p value** | **n value** |
| **2C** | Correlation  visual responses and normalized amplitude | *Spearman’s correlation* | Visual responses (%) vs normalized Δ in amplitude at 48 hours (run on bin average)  r = -0.951 | p < 0.001 | Deprived =  426 spines  16 dendritic branches |
| **2F** | Probability of locally clustered spine-types  Actual vs Shuffled | *One-Way ANOVA* | Actual vs Shuffled | p = 0.159 |  |

**Figure 2-source data 1.** Statistical comparisons for Figure 2.
