# Supplementary material for: Homeostatic regulation through strengthening of neuronal network-correlated synaptic inputs
Source: eLife. 2022 Dec 14;11:e81958. doi: 10.7554/eLife.81958 (PMC9803349; doi:10.7554/eLife.81958)
Supplement: Figure 3—source data 1. [file elife-81958-fig3-data1.docx]

| **Statistical Comparisons**  **for Figure 3** | | | **Comparison** | **Result** | |
| --- | --- | --- | --- | --- | --- |
| **Panel** | **Description** | **Test** |  | **p value** | **n value** |
| **3A** | Normalized change in amplitude of spine events for  visual spines  Control vs Deprived | *Two-Way ANOVA with post-hoc test* | Amplitude: Control vs Deprived | p = 0.004 | Deprived  Visual =  112 spines  Control  Visual =  143 spines  Deprived Unclassified =  41 spines  Control  Unclassified =  75 spines  Deprived  Network =  273 spines  Control  Network =  428 spines |
|  |  |  | -24 hrs: Con vs Dep | p = 0.733 |  |
|  |  |  | -1 hrs: Con vs Dep | p = 0.733 |  |
|  |  |  | +12 hrs: Con vs Dep | p < 0.001 |  |
|  |  |  | +24 hrs: Con vs Dep | p = 0.465 |  |
|  |  |  | +48 hrs: Con vs Dep | p = 0.016 |  |
|  |  | *One-Way*  *Repeated measures ANOVA* | Amplitude: Dep -1 hrs vs Dep 12,24,48 hrs | p = 0.152 |  |
|  |  |  | Amplitude: Con -1 hrs vs Con 12,24,48 hrs | p = 0.166 |  |
| **3B** | Normalized change in amplitude of spine events for unclassified  spines  Control vs Deprived | *Two-Way ANOVA with post-hoc test* | Amplitude: Control vs Deprived | p = 0.557 |  |
|  |  |  | -24 hrs: Con vs Dep | p = 0.373 |  |
|  |  |  | -1 hrs: Con vs Dep | p = 0.373 |  |
|  |  |  | +12 hrs: Con vs Dep | p = 0.197 |  |
|  |  |  | +24 hrs: Con vs Dep | p = 0.806 |  |
|  |  |  | +48 hrs: Con vs Dep | p = 0.835 |  |
|  |  | *One-Way*  *Repeated measures ANOVA* | Amplitude: Dep -1 hrs vs Dep 12,24,48 hrs | p = 0.683 |  |
|  |  |  | Amplitude: Con -1 hrs vs Con 12,24,48 hrs | p = 0.558 |  |
| **3C** | Normalized change in amplitude of spine events for network spines  Control vs Deprived | *Two-Way ANOVA with post-hoc test* | Amplitude: Control vs Deprived | p < 0.001 |  |
|  |  |  | -24 hrs: Con vs Dep | p = 0.841 |  |
|  |  |  | -1 hrs: Con vs Dep | p = 0.841 |  |
|  |  |  | +12 hrs: Con vs Dep | p = 0.505 |  |
|  |  |  | +24 hrs: Con vs Dep | p < 0.001 |  |
|  |  |  | +48 hrs: Con vs Dep | p < 0.001 |  |
|  |  | *One-Way*  *Repeated measures ANOVA* | Amplitude: Dep -1 hrs vs Dep 12,24,48 hrs | p = 0.008 |  |
|  |  |  | Dep -1 hrs vs +12 hrs | p = 0.556 |  |
|  |  |  | Dep -1 hrs vs +24 hrs | p = 0.015 |  |
|  |  |  | Dep -1 hrs vs +48 hrs | p = 0.045 |  |
|  |  |  | Amplitude: Con -1 hrs vs Con 12,24,48 hrs | p = 0.122 |  |
| **3D** | % of inactive visual spines  Con vs Dep | *Chi-square*  *test* | +12 hrs: Con (0.7 %) vs Dep (30.4%)  +24 hrs: Con (0.7 %) vs Dep (16.1 %)  +48 hrs: Con (1.4 %) vs Dep (16.1 %) | p < 0.001  p < 0.001  p < 0.001 |  |
| **3E** | % of inactive unclassified spines  Con vs Dep | *Chi-square*  *test* | +12 hrs: Con (1.3 %) vs Dep (14.6%)  +24 hrs: Con (5.3 %) vs Dep (14.6 %)  +48 hrs: Con (2.7 %) vs Dep (4.9 %) | p = 0.024  p = 0.228  p = 0.951 |  |
| **3F** | % of inactive network spines  Con vs Dep | *Chi-square*  *test* | +12 hrs: Con (0.2 %) vs Dep (14.7%)  +24 hrs: Con (3.0 %) vs Dep (10.3 %)  +48 hrs: Con (1.4 %) vs Dep (7.3 %) | p < 0.001  p = 0.052  p < 0.001 |  |
| **3G** | Correlation normalized frequency and amplitude | *Pearson correlation* | Visual spines (r = -0.014) | p = 0.969 |  |
| **3H** |  |  | Unclassified spines (r = 0.051) | p = 0.897 |  |
| **3I** |  |  | Network spines (r = 0.215) | p = 0.551 |  |

**Figure 3-source data 1.** Statistical comparisons for Figure 3.
