# Supplementary material for: Homeostatic regulation through strengthening of neuronal network-correlated synaptic inputs
Source: eLife. 2022 Dec 14;11:e81958. doi: 10.7554/eLife.81958 (PMC9803349; doi:10.7554/eLife.81958)
Supplement: Figure 3—figure supplement 1—source data 1. [file elife-81958-fig3-figsupp1-data1.docx]

| **Statistical Comparisons**  **for Figure 3 - figure supplement 1** | | | **Comparison** | **Result** | |
| --- | --- | --- | --- | --- | --- |
| **Panel** | **Description** | **Test** |  | **p value** | **n value** |
| **3-1A** | Normalized change in frequency of spine events for  visual spines  Control vs Deprived | *Two-Way ANOVA with post-hoc test* | Frequency: Control vs Deprived | p < 0.001 | Deprived  Visual =  112 spines  Control  Visual =  143 spines  Deprived Unclassified =  41 spines  Control  Unclassified =  75 spines  Deprived  Network =  273 spines  Control  Network =  428 spines |
|  |  |  | -24 hrs: Con vs Dep | p = 0.064 |  |
|  |  |  | -1 hrs: Con vs Dep | p = 0.064 |  |
|  |  |  | +12 hrs: Con vs Dep | p < 0.001 |  |
|  |  |  | +24 hrs: Con vs Dep | p < 0.001 |  |
|  |  |  | +48 hrs: Con vs Dep | p < 0.001 |  |
|  |  | *One-Way*  *Repeated measures ANOVA* | Frequency: Dep -1 hrs vs Dep 12,24,48 hrs | p < 0.001 |  |
|  |  |  | Dep -1 hrs vs +12 hrs | p < 0.001 |  |
|  |  |  | Dep -1 hrs vs +24 hrs | p < 0.001 |  |
|  |  |  | Dep -1 hrs vs +48 hrs | p = 0.002 |  |
|  |  |  | Frequency: Con -1 hrs vs Con 12,24,48 hrs | p = 0.056 |  |
| **3-1B** | Normalized change in frequency of spine events for unclassified spines  Control vs Deprived | *Two-Way ANOVA with post-hoc test* | Frequency: Control vs Deprived | p = 0.014 |  |
|  |  |  | -24 hrs: Con vs Dep | p = 0.227 |  |
|  |  |  | -1 hrs: Con vs Dep | p = 0.227 |  |
|  |  |  | +12 hrs: Con vs Dep | p = 0.002 |  |
|  |  |  | +24 hrs: Con vs Dep | p = 0.370 |  |
|  |  |  | +48 hrs: Con vs Dep | p = 0.141 |  |
|  |  | *One-Way*  *Repeated measures ANOVA* | Frequency: Dep -1 hrs vs Dep 12,24,48 hrs | p = 0.339 |  |
|  |  |  | Frequency: Con -1 hrs vs Con 12,24,48 hrs | p = 0.009 |  |
|  |  |  | Con -1 hrs vs +12 hrs | p = 0.055 |  |
|  |  |  | Con -1 hrs vs +24 hrs | p = 0.926 |  |
|  |  |  | Con -1 hrs vs +48 hrs | p = 0.035 |  |
| **3-1C** | Normalized change in frequency of spine events for network spines  Control vs Deprived | *Two-Way ANOVA with post-hoc test* | Frequency: Control vs Deprived | p < 0.001 |  |
|  |  |  | -24 hrs: Con vs Dep | p = 0.002 |  |
|  |  |  | -1 hrs: Con vs Dep | p = 0.002 |  |
|  |  |  | +12 hrs: Con vs Dep | p < 0.001 |  |
|  |  |  | +24 hrs: Con vs Dep | p = 0.034 |  |
|  |  |  | +48 hrs: Con vs Dep | p < 0.001 |  |
|  |  | *One-Way*  *Repeated measures ANOVA* | Frequency: Dep -1 hrs vs Dep 12,24,48 hrs | p = 0.713 |  |
|  |  |  | Frequency: Con -1 hrs vs Con 12,24,48 hrs | p < 0.001 |  |
|  |  |  | Con -1 hrs vs +12 hrs | p = 0.011 |  |
|  |  |  | Con -1 hrs vs +24 hrs | p = 0.193 |  |
|  |  |  | Con -1 hrs vs +48 hrs | p = 0.063 |  |
| **3-1D** | Normalized change in integral of spine events for  visual spines  Control vs Deprived | *Two-Way ANOVA with post-hoc test* | Integral: Control vs Deprived | p < 0.001 |  |
|  |  |  | -24 hrs: Con vs Dep | p = 0.095 |  |
|  |  |  | -1 hrs: Con vs Dep | p = 0.095 |  |
|  |  |  | +12 hrs: Con vs Dep | p < 0.001 |  |
|  |  |  | +24 hrs: Con vs Dep | p = 0.001 |  |
|  |  |  | +48 hrs: Con vs Dep | p < 0.001 |  |
|  |  | *One-Way*  *Repeated measures ANOVA* | Integral: Dep -1 hrs vs Dep 12,24,48 hrs | p = 0.007 |  |
|  |  |  | Dep -1 hrs vs +12 hrs | p = 0.006 |  |
|  |  |  | Dep -1 hrs vs +24 hrs | p = 0.020 |  |
|  |  |  | Dep -1 hrs vs +48 hrs | p = 0.006 |  |
|  |  |  | Integral: Con -1 hrs vs Con 12,24,48 hrs | p = 0.052 |  |
| **3-1E** | Normalized change in integral of spine events for  unclassified spines  Control vs Deprived | *Two-Way ANOVA with post-hoc test* | Integral: Control vs Deprived | p = 0.088 |  |
|  |  |  | -24 hrs: Con vs Dep | p = 0.243 |  |
|  |  |  | -1 hrs: Con vs Dep | p = 0.243 |  |
|  |  |  | +12 hrs: Con vs Dep | p = 0.028 |  |
|  |  |  | +24 hrs: Con vs Dep | p = 0.289 |  |
|  |  |  | +48 hrs: Con vs Dep | p = 0.576 |  |
|  |  | *One-Way*  *Repeated measures ANOVA* | Integral: Dep -1 hrs vs Dep 12,24,48 hrs | p = 0.326 |  |
|  |  |  | Integral: Con -1 hrs vs Con 12,24,48 hrs | p = 0.113 |  |
| **3-1F** | Normalized change in integral of spine events for  network spines  Control vs Deprived | *Two-Way ANOVA with post-hoc test* | Integral: Control vs Deprived | p < 0.001 |  |
|  |  |  | -24 hrs: Con vs Dep | p = 0.169 |  |
|  |  |  | -1 hrs: Con vs Dep | p = 0.169 |  |
|  |  |  | +12 hrs: Con vs Dep | p = 0.174 |  |
|  |  |  | +24 hrs: Con vs Dep | p = 0.001 |  |
|  |  |  | +48 hrs: Con vs Dep | p < 0.001 |  |
|  |  | *One-Way*  *Repeated measures ANOVA* | Integral: Dep -1 hrs vs Dep 12,24,48 hrs | p = 0.015 |  |
|  |  |  | Dep -1 hrs vs +12 hrs | p = 0.081 |  |
|  |  |  | Dep -1 hrs vs +24 hrs | p = 0.075 |  |
|  |  |  | Dep -1 hrs vs +48 hrs | p = 0.004 |  |
|  |  |  | Integral: Con -1 hrs vs Con 12,24,48 hrs | p = 0.004 |  |
|  |  |  | Con -1 hrs vs +12 hrs | p = 0.614 |  |
|  |  |  | Con -1 hrs vs +24 hrs | p = 0.012 |  |
|  |  |  | Con -1 hrs vs +48 hrs | p = 0.620 |  |

**Figure 3-source data 2.** Statistical comparisons for Figure 3 - figure supplement 1.
