# Supplementary material for: Homeostatic regulation through strengthening of neuronal network-correlated synaptic inputs
Source: eLife. 2022 Dec 14;11:e81958. doi: 10.7554/eLife.81958 (PMC9803349; doi:10.7554/eLife.81958)
Supplement: Figure 4—source data 1. [file elife-81958-fig4-data1.docx]

| **Statistical Comparisons**  **for Figure 4** | | | **Comparison** | **Result** | |
| --- | --- | --- | --- | --- | --- |
| **Panel** | **Description** | **Test** |  | **p value** | **n value** |
| **4C** | Normalized change in amplitude of spine events following visual deprivation  Deprived  vs  Control  vs  Deprived + TNF-α inhibitor | *Two-Way ANOVA with post-hoc test* | Control vs Deprived | p < 0.001 | Deprived = 1050 spines  Control = 615 spines  Deprived +  TNF-α inhibitor = 629 spines |
|  |  |  | Deprived vs Deprived+TNF-α inhibitor | p < 0.001 |  |
|  |  |  | Control vs Deprived+TNF-α inhibitor | p = 0.043 |  |
|  |  |  | -1 hrs: Con vs Dep | p = 0.029 |  |
|  |  |  | -1 hrs: Dep vs Dep+TNF-α inhibitor | p = 0.808 |  |
|  |  |  | -1 hrs: Con vs Dep+TNF-α inhibitor | p = 0.061 |  |
|  |  |  | 0 hrs: Con vs Dep | p = 0.029 |  |
|  |  |  | 0 hrs: Dep vs Dep+TNF-α inhibitor | p = 0.808 |  |
|  |  |  | 0 hrs: Con vs Dep+TNF-α inhibitor | p = 0.061 |  |
|  |  |  | +48 hrs: Con vs Dep | p < 0.001 |  |
|  |  |  | +48 hrs: Dep vs Dep+TNF-α inhibitor | p < 0.001 |  |
|  |  |  | +48 hrs: Con vs Dep+TNF-α inhibitor | p < 0.001 |  |
| **4D** | Normalized change in amplitude of spine events of different groups (Visual, Auditory & Network)  following visual deprivation  Deprived  vs  Control | *Two-Way ANOVA with post-hoc test* | -1 hrs: Dep Network vs Con Visual | p = 0.595 |  |
|  |  |  | 0 hrs: Dep Network vs Con Visual | p = 0.595 |  |
|  |  |  | +48 hrs: Dep Network vs Con Visual | p = 0.006 |  |
|  |  |  | -1 hrs: Dep Network vs Dep Visual | p = 0.908 |  |
|  |  |  | 0 hrs: Dep Network vs Dep Visual | p = 0.908 |  |
|  |  |  | +48 hrs: Dep Network vs Dep Visual | p < 0.001 |  |
|  |  |  | -1 hrs: Dep Visual vs Con Visual | p = 0.825 |  |
|  |  |  | 0 hrs: Dep Visual vs Con Visual | p = 0.825 |  |
|  |  |  | +48 hrs: Dep Visual vs Con Visual | p = 0.878 |  |
| **4E** |  |  | -1 hrs: Dep Network vs Con Auditory | p = 0.876 |  |
|  |  |  | 0 hrs: Dep Network vs Con Auditory | p = 0.876 |  |
|  |  |  | +48 hrs: Dep Network vs Con Auditory | p < 0.001 |  |
|  |  |  | -1 hrs: Dep Network vs Dep Auditory | p = 0.813 |  |
|  |  |  | 0 hrs: Dep Network vs Dep Auditory | p = 0.813 |  |
|  |  |  | +48 hrs: Dep Network vs Dep Auditory | p < 0.001 |  |
|  |  |  | -1 hrs: Dep Auditory vs Con Auditory | p = 0.642 |  |
|  |  |  | 0 hrs: Dep Auditory vs Con Auditory | p = 0.642 |  |
|  |  |  | +48 hrs: Dep Auditory vs Con Auditory | p = 0.953 |  |
| **4G** | Normalized change in amplitude of spine events following auditory deprivation  Deprived vs  Control vs  Deprived + TNF-α inhibitor | *Two-Way ANOVA with post-hoc test* | Control vs Deprived | p = 0.012 | Deprived = 801 spines  Control = 572 spines  Deprived +  TNF-α inhibitor = 837 spines |
|  |  |  | Deprived vs Deprived+TNF-α inhibitor | p < 0.001 |  |
|  |  |  | Control vs Deprived+TNF-α inhibitor | p = 0.051 |  |
|  |  |  | -1 hrs: Con vs Dep | p = 0.048 |  |
|  |  |  | -1 hrs: Dep vs Dep+TNF-α inhibitor | p = 0.196 |  |
|  |  |  | -1 hrs: Con vs Dep+TNF-α inhibitor | p = 0.335 |  |
|  |  |  | 0 hrs: Con vs Dep | p = 0.048 |  |
|  |  |  | 0 hrs: Dep vs Dep+TNF-α inhibitor | p = 0.196 |  |
|  |  |  | 0 hrs: Con vs Dep+TNF-α inhibitor | p = 0.335 |  |
|  |  |  | +48 hrs: Con vs Dep | p < 0.001 |  |
|  |  |  | +48 hrs: Dep vs Dep+TNF-α inhibitor | p < 0.001 |  |
|  |  |  | +48 hrs: Con vs Dep+TNF-α inhibitor | p < 0.001 |  |
| **4H** | Normalized change in amplitude of spine events of different groups (Visual, Auditory & Network)  following auditory deprivation  Deprived  vs  Control | *Two-Way ANOVA with post-hoc test* | -1 hrs: Dep Network vs Con Visual | p = 0.053 |  |
|  |  |  | 0 hrs: Dep Network vs Con Visual | p = 0.053 |  |
|  |  |  | +48 hrs: Dep Network vs Con Visual | p < 0.001 |  |
|  |  |  | -1 hrs: Dep Network vs Dep Visual | p = 0.813 |  |
|  |  |  | 0 hrs: Dep Network vs Dep Visual | p = 0.813 |  |
|  |  |  | +48 hrs: Dep Network vs Dep Visual | p < 0.001 |  |
|  |  |  | -1 hrs: Dep Visual vs Con Visual | p = 0.690 |  |
|  |  |  | 0 hrs: Dep Visual vs Con Visual | p = 0.690 |  |
|  |  |  | +48 hrs: Dep Visual vs Con Visual | p = 0.203 |  |
| **4I** |  |  | -1 hrs: Dep Network vs Con Auditory | p = 0.782 |  |
|  |  |  | 0 hrs: Dep Network vs Con Auditory | p = 0.782 |  |
|  |  |  | +48 hrs: Dep Network vs Con Auditory | p = 0.002 |  |
|  |  |  | -1 hrs: Dep Network vs Dep Auditory | p = 0.919 |  |
|  |  |  | 0 hrs: Dep Network vs Dep Auditory | p = 0.919 |  |
|  |  |  | +48 hrs: Dep Network vs Dep Auditory | p < 0.001 |  |
|  |  |  | -1 hrs: Dep Auditory vs Con Auditory | p = 0.966 |  |
|  |  |  | 0 hrs: Dep Auditory vs Con Auditory | p = 0.966 |  |
|  |  |  | +48 hrs: Dep Auditory vs Con Auditory | p = 0.862 |  |
| **4J** | Global integral  to visual or auditory stimuli | *Paired t-test* | Visual: 0 hrs vs +48 hrs | p < 0.001 |  |
|  |  |  | Auditory: 0 hrs vs +48 hrs | p < 0.001 |  |
| **4K** | Δ branch activity vs  Δ spine activity | *Pearson correlation* | Visual spines: r = 0.56 | p = 0.098 |  |
| **4L** |  |  | Auditory spines: r = 0.30 | p = 0.439 |  |
| **4M** |  |  | Network spines: r = 0.93 | p < 0.001 |  |

**Figure 4-source data 1.** Statistical comparisons for Figure 4.
