# Supplementary material for: Homeostatic regulation through strengthening of neuronal network-correlated synaptic inputs
Source: eLife. 2022 Dec 14;11:e81958. doi: 10.7554/eLife.81958 (PMC9803349; doi:10.7554/eLife.81958)
Supplement: Figure 4—figure supplement 1—source data 1. [file elife-81958-fig4-figsupp1-data1.docx]

| **Statistical Comparisons**  **for Figure 4 - figure supplement 1** | | | **Comparison** | **Result** | |
| --- | --- | --- | --- | --- | --- |
| **Panel** | **Description** | **Test** |  | **p value** | **n value** |
| **4-1B** | Normalized change in amplitude of spine events following visual deprivation  Deprived Network  vs  Control Network  vs  Deprived + TNF-α inhibitor Network | *Two-Way ANOVA with post-hoc test* | Control Network vs Deprived Network | p = 0.005 | Deprived Unclassified  = 86 spines  Control  Unclassified = 54 spines  Deprived  Network =  599 spines  Control  Network =  345 spines  Deprived Network +TNF-α inhibitor = 368 spines |
|  |  |  | Deprived Network vs Deprived+TNF-α inhibitor Network | p < 0.001 |  |
|  |  |  | Control Network vs Deprived+TNF-α inhibitor Network | p = 0.214 |  |
|  |  |  | -1 hrs: Con Net vs Dep Net | p = 0.044 |  |
|  |  |  | -1 hrs: Dep Net vs Dep+TNF-α inhibitor Net | p = 0.646 |  |
|  |  |  | -1 hrs: Con Net vs Dep+TNF-α inhibitor Net | p = 0.027 |  |
|  |  |  | 0 hrs: Con Net vs Dep Net | p = 0.044 |  |
|  |  |  | 0 hrs: Dep Net vs Dep+TNF-α inhibitor Net | p = 0.646 |  |
|  |  |  | 0 hrs: Con Net vs Dep+TNF-α inhibitor Net | p = 0.027 |  |
|  |  |  | +48 hrs: Con Net vs Dep Net | p < 0.001 |  |
|  |  |  | +48 hrs: Dep Net vs Dep+TNF-α inhibitor Net | p < 0.001 |  |
|  |  |  | +48 hrs: Con Net vs Dep+TNF-α inhibitor Net | p = 0.031 |  |
| **4-1C** | Normalized change in amplitude of spine events following visual deprivation  Deprived Unclassified  vs  Control  Unclassified  vs  Deprived Network | *Two-Way ANOVA with post-hoc test* |  |  |  |
|  |  |  | -1 hrs: Dep Network vs Con Unclassified | p = 0.925 |  |
|  |  |  | 0 hrs: Dep Network vs Con Unclassified | p = 0.925 |  |
|  |  |  | +48 hrs: Dep Network vs Con Unclassified | p = 0.007 |  |
|  |  |  |  |  |  |
|  |  |  | -1 hrs: Dep Network vs Dep Unclassified | p = 0.622 |  |
|  |  |  | 0 hrs: Dep Network vs Dep Unclassified | p = 0.622 |  |
|  |  |  | +48 hrs: Dep Network vs Dep Unclassified | p = 0.008 |  |
|  |  |  |  |  |  |
|  |  |  | -1 hrs: Dep Unclassified vs Con Unclassified | p = 0.775 |  |
|  |  |  | 0 hrs: Dep Unclassified vs Con Unclassified | p = 0.775 |  |
|  |  |  | +48hrs: Dep Unclassified vs Con Unclassified | p = 0.933 |  |
| **4-1E** | Normalized change in amplitude of spine events following auditory deprivation  Deprived Network  vs  Control Network  vs  Deprived + TNF-α inhibitor Network | *Two-Way ANOVA with post-hoc test* | Control Network vs Deprived Network | p = 0.011 | Deprived Unclassified = 62 spines  Control Unclassified = 43 spines  Deprived Network = 464 spines  Control Network = 336 spines  Deprived Network +TNF-α inhibitor = 506 spines |
|  |  |  | Deprived Network vs Deprived+TNF-α inhibitor Network | p < 0.001 |  |
|  |  |  | Control Network vs Deprived+TNF-α inhibitor Network | p = 0.009 |  |
|  |  |  | -1 hrs: Con Net vs Dep Net | p = 0.007 |  |
|  |  |  | -1 hrs: Dep Net vs Dep+TNF-α inhibitor Net | p = 0.017 |  |
|  |  |  | -1 hrs: Con Net vs Dep+TNF-α inhibitor Net | p = 0.460 |  |
|  |  |  | 0 hrs: Con Net vs Dep Net | p = 0.007 |  |
|  |  |  | 0 hrs: Dep Net vs Dep+TNF-α inhibitor Net | p = 0.017 |  |
|  |  |  | 0 hrs: Con Net vs Dep+TNF-α inhibitor Net | p = 0.460 |  |
|  |  |  | +48 hrs: Con Net vs Dep Net | p < 0.001 |  |
|  |  |  | +48 hrs: Dep Net vs Dep+TNF-α inhibitor Net | p < 0.001 |  |
|  |  |  | +48 hrs: Con Net vs Dep+TNF-α inhibitor Net | p < 0.001 |  |
| **4-1F** | Normalized change in amplitude of spine events following auditory deprivation  Deprived Unclassified  vs  Control  Unclassified  vs  Deprived Network | *Two-Way ANOVA with post-hoc test* |  |  |  |
|  |  |  | -1 hrs: Dep Network vs Con Unclassified | p = 0.602 |  |
|  |  |  | 0 hrs: Dep Network vs Con Unclassified | p = 0.602 |  |
|  |  |  | +48 hrs: Dep Network vs Con Unclassified | p = 0.004 |  |
|  |  |  |  |  |  |
|  |  |  | -1 hrs: Dep Network vs Dep Unclassified | p = 0.753 |  |
|  |  |  | 0 hrs: Dep Network vs Dep Unclassified | p = 0.753 |  |
|  |  |  | +48 hrs: Dep Network vs Dep Unclassified | p = 0.003 |  |
|  |  |  |  |  |  |
|  |  |  | -1 hrs: Dep Unclassified vs Con Unclassified | p = 0.821 |  |
|  |  |  | 0 hrs: Dep Unclassified vs Con Unclassified | p = 0.821 |  |
|  |  |  | +48hrs: Dep Unclassified vs Con Unclassified | p = 0.752 |  |
|  | Change in OSI at 48 hours for spines & dendrites  Deprived vs Control | *t-test* | Spines: Control vs Deprived | p = 0.956 |  |
|  |  |  | Dendrites: Control vs Deprived | p = 0.461 |  |

**Figure 4-source data 2.** Statistical comparisons for Figure 4 - figure supplement 1.
